# Genome Sequence of *Gordonia* Phage BetterKatz

**DOI:** 10.1128/genomeA.00590-16

**Published:** 2016-08-11

**Authors:** Welkin H. Pope, Emily N. Berryman, Kaitlyn M. Forrest, Lilliana McHale, Anthony T. Wertz, Zenas Zhuang, Naomi S. Kasturiarachi, Catherine A. Pressimone, Johnathon G. Schiebel, Emily C. Furbee, Sarah R. Grubb, Marcie H. Warner, Matthew T. Montgomery, Rebecca A. Garlena, Daniel A. Russell, Deborah Jacobs-Sera, Graham F. Hatfull

**Affiliations:** Department of Biological Sciences, University of Pittsburgh, Pittsburgh, Pennsylvania, USA

## Abstract

BetterKatz is a bacteriophage isolated from a soil sample collected in Pittsburgh, Pennsylvania using the host *Gordonia terrae* 3612. BetterKatz’s genome is 50,636 bp long and contains 75 predicted protein-coding genes, 35 of which have been assigned putative functions. BetterKatz is not closely related to other sequenced *Gordonia* phages.

## GENOME ANNOUNCEMENT

*Gordonia* spp. are common environmental inhabitants (1) and several bacteriophages have been isolated using *Gordonia* hosts (2–6). To understand the genetic diversity of *Gordonia* phages, the integrated research-education Science Education Alliance-Phage Hunters Advancing Genomic and Evolutionary Science (SEA-PHAGES) program is using *Gordonia terrae* 3612 to isolate and genomically characterize bacteriophages (7). Phage BetterKatz was recovered from a soil sample from Pittsburgh, PA by direct plating of filtered soil extract on a lawn of *G. terrae*; it was plaque purified, amplified, and viral dsDNA was extracted. BetterKatz virions have a siphoviral morphology with an isometric head, and a flexible tail 220 nm in length.

BetterKatz was sequenced using the Illumina MiSeq platform using 140 bp single-end reads and assembled using Newbler to yield a single major contig of 50,636 bp with an average coverage of 247-fold. The genome has defined ends with 10 base 3′ single stranded DNA extensions (5′-TGCCGCGGTA) and is 67.1% G+C, similar to its host (67.8%). BetterKatz does not share extensive nucleotide sequence similarity to other sequenced phages or prophages, although there are two segments spanning approximately 10 kbp—corresponding to virion structural genes—with similarity to a putative prophage in *Gordonia* sp. KTR9 (8) that is integrated at an *attB* site overlapping a tRNA^ala^ gene (KTR9_RS07590).

Seventy-five BetterKatz protein-coding genes were predicted using Glimmer and Genemark (9, 10) and putative functions were assigned using BLASTP, HHpred, and Phamerator (11, 12); no tRNA genes are predicted using Aragorn (13). All are transcribed rightwards with the exception of five genes—including a tyrosine-integrase and the immunity repressor—near the center of the genome. The attachment site (*attP*) is located immediately downstream of *int* (*39*) and BetterKatz is predicted to integrate into the same *attB* site overlapping a tRNA^ala^ gene, where prophages lie in both *Gordonia* sp. KTR9 and *Gordonia bronchialis* DSM 43427 (14). The genes in the left arm are predominantly virion structure and assembly genes, and several genes in the right arm encode putative DNA metabolism functions including a DNA primase, a DNA methylase, and an exonuclease. We note that 31 of the predicted genes have no amino acid sequence similarity to other actinobacteriophage-encoded proteins in a data set of over 150,000 genes.

The lysis cassette in BetterKatz is located immediately downstream of the virion tail genes and there are two genes with predicted endolysin functions, gp29 that encodes a cysteine protease-like protein and gp30 encoding a glycoside hydrolase. The product of gene *31* has three predicted transmembrane domains and is the likely holin, although gp32 is also a putative membrane protein with four transmembrane domains and may also play a role in lysis. Immediately to the right of the lysis cassette is a leftwards-transcribed HicAB-like toxin-antitoxin system (15). A putative transcription promoter is located upstream of the toxin gene (*35*) and a region of dyad symmetry overlaps the putative −10 motif to which the antitoxin (gp34) may bind to regulate TA transcription.

### Accession number(s).

The BetterKatz genome sequence is available from GenBank under accession number KU963261.
